# Europe PMC in 2023

**DOI:** 10.1093/nar/gkad1085

**Published:** 2023-11-22

**Authors:** Summer Rosonovski, Maria Levchenko, Rajat Bhatnagar, Umamageswari Chandrasekaran, Lynne Faulk, Islam Hassan, Matt Jeffryes, Syed Irtaza Mubashar, Maaly Nassar, Madhumiethaa Jayaprabha Palanisamy, Michael Parkin, Jagadeeswararao Poluru, Frances Rogers, Shyamasree Saha, Mohamed Selim, Zunaira Shafique, Michele Ide-Smith, David Stephenson, Santosh Tirunagari, Aravind Venkatesan, Lijun Xing, Melissa Harrison

**Affiliations:** Literature Services, EMBL-EBI, Wellcome Trust Genome Campus, Cambridge, UK; Literature Services, EMBL-EBI, Wellcome Trust Genome Campus, Cambridge, UK; Literature Services, EMBL-EBI, Wellcome Trust Genome Campus, Cambridge, UK; Literature Services, EMBL-EBI, Wellcome Trust Genome Campus, Cambridge, UK; Literature Services, EMBL-EBI, Wellcome Trust Genome Campus, Cambridge, UK; Literature Services, EMBL-EBI, Wellcome Trust Genome Campus, Cambridge, UK; Literature Services, EMBL-EBI, Wellcome Trust Genome Campus, Cambridge, UK; Literature Services, EMBL-EBI, Wellcome Trust Genome Campus, Cambridge, UK; Literature Services, EMBL-EBI, Wellcome Trust Genome Campus, Cambridge, UK; Literature Services, EMBL-EBI, Wellcome Trust Genome Campus, Cambridge, UK; Literature Services, EMBL-EBI, Wellcome Trust Genome Campus, Cambridge, UK; Literature Services, EMBL-EBI, Wellcome Trust Genome Campus, Cambridge, UK; Literature Services, EMBL-EBI, Wellcome Trust Genome Campus, Cambridge, UK; Literature Services, EMBL-EBI, Wellcome Trust Genome Campus, Cambridge, UK; Literature Services, EMBL-EBI, Wellcome Trust Genome Campus, Cambridge, UK; Literature Services, EMBL-EBI, Wellcome Trust Genome Campus, Cambridge, UK; Literature Services, EMBL-EBI, Wellcome Trust Genome Campus, Cambridge, UK; Literature Services, EMBL-EBI, Wellcome Trust Genome Campus, Cambridge, UK; Literature Services, EMBL-EBI, Wellcome Trust Genome Campus, Cambridge, UK; Literature Services, EMBL-EBI, Wellcome Trust Genome Campus, Cambridge, UK; Literature Services, EMBL-EBI, Wellcome Trust Genome Campus, Cambridge, UK; Literature Services, EMBL-EBI, Wellcome Trust Genome Campus, Cambridge, UK

## Abstract

Europe PMC (https://europepmc.org/) is an open access database of life science journal articles and preprints, which contains over 42 million abstracts and over 9 million full text articles accessible via the website, APIs and bulk download. This publication outlines new developments to the Europe PMC platform since the last database update in 2020 (1) and focuses on five main areas. (i) Improving discoverability, reproducibility and trust in preprints by indexing new preprint content, enriching preprint metadata and identifying withdrawn and removed preprints. (ii) Enhancing support for text and data mining by expanding the types of annotations provided and developing the Europe PMC Annotations Corpus, which can be used to train machine learning models to increase their accuracy and precision. (iii) Developing the Article Status Monitor tool and email alerts, to notify users about new articles and updates to existing records. (iv) Positioning Europe PMC as an open scholarly infrastructure through increasing the portion of open source core software, improving sustainability and accessibility of the service.

## Graphical Abstract

**Graphical Abstract:**
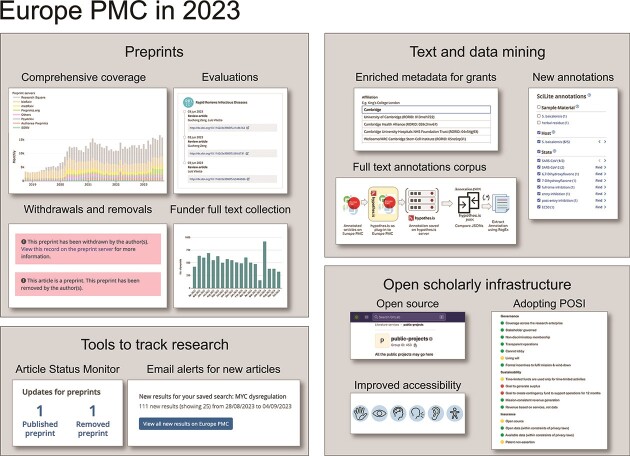

## Introduction

Europe PMC is a freely available, life science literature database hosted by EMBL’s European Bioinformatics Institute (EMBL-EBI). It is an ELIXIR Core Data Resource (2) and a Global Core Biodata Resource (https://globalbiodata.org/what-we-do/global-core-biodata-resources/list-of-current-global-core-biodata-resources/), endorsed and supported by 37 international research funders as their dedicated open access repository (https://europepmc.org/Funders/). The content scope includes abstracts and metadata for journal articles from PubMed (3), AGRICOLA (https://www.nal.usda.gov/agricola) and other sources, as well as full text publications through a collaboration with PubMed Central (PMC) archive (3). As of 15 September 2023, Europe PMC has indexed over 42 million abstracts and over 9 million full text articles. Where full text is not available in Europe PMC, links to free full text for over 13 million publications can be accessed via Unpaywall (https://unpaywall.org/). In addition, Europe PMC indexes abstracts and metadata for preprints from 31 different preprint servers (https://europepmc.org/Preprints#preprint-servers), with over 650 000 preprints indexed, of which 53 624 has full text available on the platform at the time of writing. The content in Europe PMC is updated daily and can be explored and accessed using the Europe PMC website (https://europepmc.org/) and programmatically via RESTful APIs (https://europepmc.org/RestfulWebService) and FTP bulk downloads (https://europepmc.org/downloads).

To facilitate information discovery and enhance content, Europe PMC provides innovative features and tools, such as the Grant Finder (https://europepmc.org/grantfinder), ORCID claiming (https://europepmc.org/orcid/import), text-mined biological annotations (https://europepmc.org/Annotations) and more. Publications in Europe PMC are enriched in a number of ways, including links to data in over 60 life science databases, citations, funding, protocols and peer review materials.

This article details the most significant updates to Europe PMC since our previous report in 2020 (1). These changes focus on five overarching themes, including building trust in preprints, enrichment of articles, keeping up to date with research, improved accessibility and user experience, and the sustainability of Europe PMC.

Europe PMC has long supported preprints as an effective means of research communication. To improve preprint discoverability, Europe PMC has significantly expanded its preprint coverage, increasing the number of indexed preprint servers and indexing full text for Europe PMC funder preprints. For better transparency around preprint inclusion we have reviewed and updated the Europe PMC preprint indexing guidelines. Finally, we have implemented a mechanism to identify withdrawn and removed preprints.

Europe PMC uses text-mining and machine learning techniques to support evidence extraction from the research literature. In addition to its own text-mining pipeline, Europe PMC operates an annotations platform that accepts and publishes text-mining outputs from the wider community. Resulting annotations for biological entities and relations are made available on the Europe PMC website through the SciLite tool (https://europepmc.org/Annotations) and programmatically via the Annotations API (https://europepmc.org/AnnotationsApi). Since the last update, Europe PMC has continued to grow its text-mining platform with new types of annotations that now include metagenomics and COVID-19 terms. To improve annotation accuracy and precision Europe PMC has developed an open source gold standard dataset to train machine learning models for entity extraction. The dataset consists of 300 full text articles with human-annotated mentions of gene/protein, disease and organism concepts. This annotated corpus can be used by natural language processing tools to support advancements in the field.

To help users keep up to date with the current research literature, Europe PMC has introduced email alerts. This feature can notify users about new results for specific search terms and covers both journal articles and preprints. Email alerts can be used to track topics, authors, journals, or even citations for publications or datasets. Along with new discoveries, users can follow updates to existing publications and preprints with the new Article Status Monitor tool (https://europepmc.org/ArticleStatusMonitor) developed by Europe PMC. This tool retrieves various status updates, including retractions, withdrawals, new and published versions.

Finally, we describe our efforts to improve sustainability, accessibility and open sourcing code for core software to align with principles of open scholarly infrastructure (4).

## Improving discoverability, reuse and trust in preprints

### Preprint coverage

**Figure 1.**
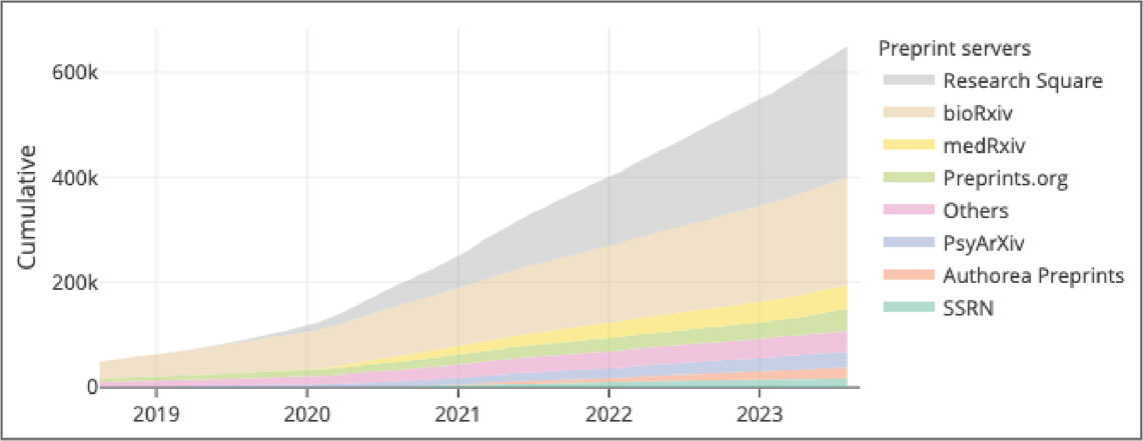
Preprints in Europe PMC (accessed August 2023).

Preprints are complete scientific manuscripts that have not undergone journal-organised peer review and are uploaded by the authors to a public server. There are multiple benefits of preprints: they are free to post and read, publicly available within days of posting, establish priority of scientific work, and allow rapid feedback to the author (https://asapbio.org/biopreprints2020-survey-initial-results) (5). Europe PMC is a long-standing supporter of preprints, and began indexing preprint abstracts from 9 preprint platforms in 2018 (https://blog.europepmc.org/2018/07/preprints-in-europe-pmc-reducing-friction-for-discoverability.html). Since then, Europe PMC has continued to broaden its preprint coverage, and now includes 31 preprint servers, bringing the total number of indexed preprints to over 650 000 as of 15 September 2023 (Figure 1 and Table 1). To offer comprehensive preprint search Europe PMC regularly monitors the preprint landscape for new and emerging servers to be added to the database (https://docs.google.com/spreadsheets/d/1ZiCUuKNse8dwHRFAyhFsZsl6kG0Fkgaj5gttdwdVZEM/edit#gid=1016151070).

**Table 1.** Europe PMC preprint coverage from indexed preprint servers (accessed 15 September 2023)

Preprint server	Year preprint server content was added to	Preprint coverage			Number of preprints indexed
	Europe PMC	Metadata/abstracts	COVID-19 full text	Funder full text	
Access Microbiology	2022	☑			224
agriRxiv	2022	☑			260
AIJR Preprints	2023	☑			125
ARPHA Preprints	2022	☑			428
arXiv	2021		☑		7666
Authorea Preprints	2020	☑	☑		21 458
Beilstein Archives	2020	☑			529
BioHackrXiv	2021	☑			54
bioRxiv	2018	☑	☑	CC-licensed only	206 666
ChemRxiv	2018	☑	☑		8950
EcoEvoRxiv	2022	☑			303
Emerald Open Research	2019	☑			121
F1000 Research	2018	☑			8173
Gates Open Research	2018	☑			458
Health Open Research	2022	☑			16
HRB Open Research	2018	☑			420
MedEdPublish	2018	☑			1697
medRxiv	2019	☑	☑	CC-licensed only	45 811
MNI Open Research	2018	☑			13
NIHR Open Research	2021	☑			69
Open Research Africa	2022	☑			36
Open Research Europe	2021	☑			442
PaleorXiv	2023	☑			143
peerJ Preprints	2018	☑			5068
Preprints.org	2018	☑	☑		43 413
PsyArXiv	2021	☑	☑		29 665
Qeios	2023	☑			1090
Research Square	2020	☑	☑	☑	253 475
SciELO Preprints	2022	☑	☑		1893
SSRN	2020		☑		17 188
Wellcome Open Research	2018	☑			1887

### Preprint indexing criteria

To safeguard the quality of preprint content in Europe PMC and ensure transparency of preprint selection criteria, Europe PMC reviewed and revised its set of preprint indexing guidelines in March 2023 (https://europepmc.org/Preprints#preprint-criteria). The current guidelines require all preprint content to be freely available with no barriers to access. Indexed servers must contain a significant proportion of life science or interdisciplinary subjects. To build trust in preprints, indexed servers should have a screening procedure and provide a public statement on policies regarding plagiarism, misconduct and competing interests. Lastly, to set up the ingest process Europe PMC requires the server to contain a minimum of 30 preprints and for the preprint metadata to be available in a machine readable format. Preprint metadata requirements are shown in Table 2.

**Table 2.** Preprint metadata requirements for inclusion of a preprint server in Europe PMC as part of the indexing guidelines

Metadata element	Requirement for indexing
Preprint identifier (Crossref DOI required)	Essential
Preprint title	Essential
Author names	Essential
Abstracts	Essential
Publication date	Essential
Author affiliations	Desired
Links to peer-reviewed versions	Desired
Licencing	Desired
Funding	Desired
Version information	Desired
Withdrawal/removal status	Desired

### Full text preprint collection

In 2020, to facilitate discovery of COVID-19 research during the coronavirus pandemic, Europe PMC started indexing the full text of COVID-19 preprints, increasing their visibility and reach (1). As of 15 September 2023, there are over 65 000 full text COVID-19 preprints in Europe PMC accessible via the website, APIs, or bulk downloads. In 2022, Europe PMC expanded the full text preprint collection to include preprints supported by at least one of Europe PMC funders. Relevant preprints that have a Creative Commons licence (https://creativecommons.org/share-your-work/cclicenses/) are identified based on the funding acknowledgement text within the preprint full text. At present, Europe PMC funder preprints are selected from medRxiv (https://www.medrxiv.org/), bioRxiv (https://www.biorxiv.org/) and Research Square (https://www.researchsquare.com/), with plans to extend the initiative to other servers in the future (6). Indexing of preprint full text in Europe PMC improves discoverability, because preprints are surfaced alongside journal published articles in Europe PMC search results if the search keywords are found beyond the abstract and metadata. It also enables advanced search options, for example limiting search to specific sections of the preprint, such as Figures or Methods. Availability of the preprint full text in a machine-readable format, such as JATS XML, supports text and data mining. This allows Europe PMC to identify data accessions and DOIs in the preprint full text, linking preprints to underlying data, therefore supporting open data sharing and improving reproducibility of science reported in preprints. Finally, programmatic access to machine-readable preprint full text supports the wider analysis of literature, contributing to the acceleration of scientific research.

### Handling preprint withdrawals and removals

**Figure 2.**
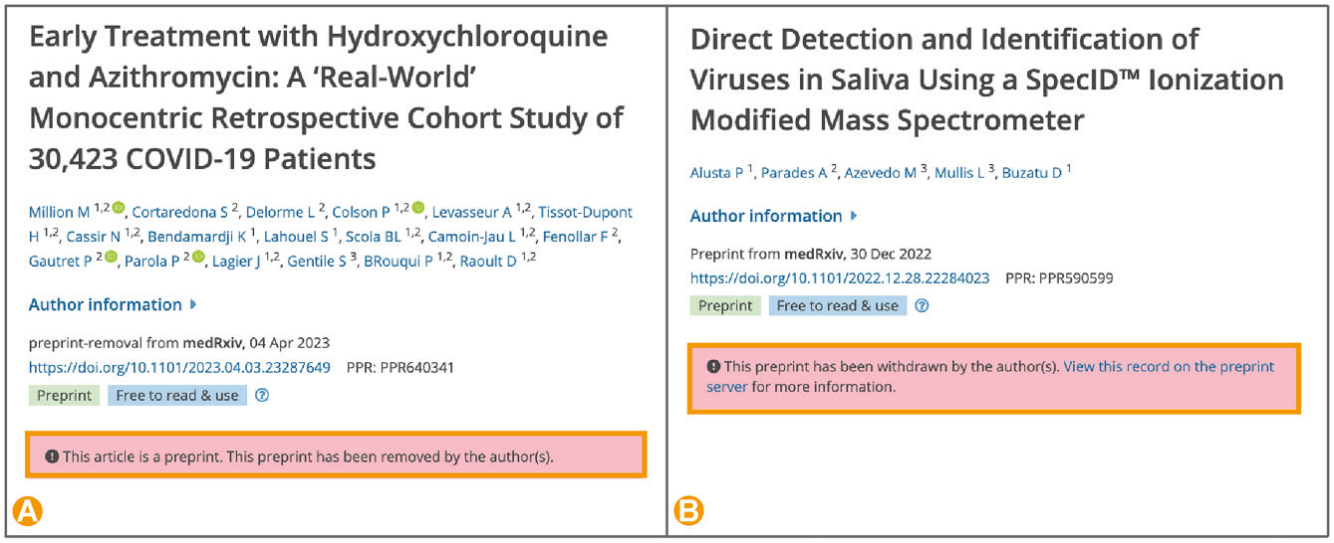
Preprint article page view in Europe PMC with (**A**) PPR640341 removal notice highlighted and (**B**) PPR590599 withdrawal notice highlighted.

One advantage of preprints is the ability to post new versions, allowing researchers to improve or correct their manuscript. In some instances authors may choose to withdraw or remove their preprint rather than update it with a new version, for example due to incorrect data or data interpretation, erroneous posting, or for legal reasons. Preprint withdrawals are managed similarly to retractions of journal articles. The preprint remains available but is supplemented with a withdrawal notice. The withdrawal notice explains to readers that the preprint should no longer be considered as a scientific record, and sometimes provides the reasons for withdrawal. In the case of preprint removals the preprint content is no longer available and is substituted with a removal notice (7). To improve transparency and trust in preprints, Europe PMC now displays prominent banners for withdrawn and removed preprints (Figure 2) (8). Withdrawn and removed preprints can be searched using respective PUB_TYPE:‘preprint-withdrawal’ and PUB_TYPE:‘preprint-removal’ search syntax. Notably, only full text preprints can be identified as withdrawn or removed in Europe PMC, as currently there is no centralised way to collect this information from preprint servers. We rely on a semi-automated process to track preprint withdrawal or removal statuses. There are also some cases when preprints are deleted entirely, with the URL resolving to a 404 page. In this case they will also be deleted from Europe PMC records to reflect the state of the preprint on the server. Europe PMC encourages preprint servers to preserve as much of the scholarly record as possible and instead change the abstract of the preprint to reflect the content has been deleted, ideally with reasoning.

### Preprint evaluations

Since preprints lack the traditional approval provided by journal peer review, there is a growing need for the scientific community to find new ways to evaluate preprinted research. To help address this, Europe PMC has developed an API that returns peer review reports, referred to as evaluations in the API, linked to a preprint: https://europepmc.org/RestfulWebService (/evaluations/{source}/{id}). Metadata for preprint reviews are retrieved from Sciety (https://sciety.org/) using the community-endorsed DocMaps framework (https://docmaps.knowledgefutures.org/pub/sgkf1pqa/release/7#:∼:text=DocMaps%20is%20a%20framework%20for,the%20consumer%20of%20the%20DocMap). Future plans include adding links to preprint evaluations currently not available in Sciety, using data available via Crossref (https://www.crossref.org/) and DataCite (https://datacite.org/).

### Preprints as first class research outputs

Europe PMC aims to establish preprints as first class research outputs. Until recently preprints were largely missing from the Europe PMC citation network as a source of reference lists. The citation network consists of reciprocal links between citing and cited articles in Europe PMC and provides the source of metadata for the ‘Cited by’ feature, citation counts, as well as /citations and /references modules of the Articles RESTful API (https://europepmc.org/RestfulWebService). To ensure that preprint handling is consistent with journal-published articles, Europe PMC now provides links from references of the full text preprints to cited journal publications in Europe PMC. This allows users to easily follow preprint citations, and with more citations available extends the utility and completeness of the citation network itself. In another example, Europe PMC worked with the ORCID organisation (https://orcid.org/) to include the Europe PMC identifier for preprints, allowing Europe PMC users to claim preprints to their ORCID record more easily and enabling hyperlinking back to the preprint page in Europe PMC. In the future Europe PMC will continue to work closely with the preprints community on standards for preprints, to improve discoverability, reusability and transparency for preprints.

### Support for text and data mining

With an increasing number of life science articles published each year, researchers are facing unprecedented levels of information overload. Text-mining approaches can support evidence synthesis by automating literature analysis. Europe PMC uses text-mining to identify relevant biological concepts, such as chemicals, organisms, or gene/protein names in research articles and preprints. In addition to its own text-mining pipeline, Europe PMC runs a community platform open for contributions from other text-mining groups. As a result, over 2 billion text-mined annotations for biological entities and relations are available via a dedicated Annotations RESTful API (https://europepmc.org/AnnotationsApi) and SciLite Annotations tool (9) as shown in Table 3. SciLite can be used to find and highlight relevant terms, including data accession numbers, cell lines, experimental methods, gene-disease relations and more, in the abstract and full text of articles and preprints with appropriate licence.

**Table 3.** Text-mined annotations available in Europe PMC

Annotation provider	Annotation type	Number of annotations in Europe PMC
Europe PMC	Database accessions	9 857 709
	Resource names	1 623 794
	Gene/protein names	519 380 726
	Organisms	276 185 000
	Diseases	311 375 453
	Chemicals	360 373 053
	Gene ontology terms	241 708 255
	Experimental methods	82 913 165
Metagenomics	Sample-material	807 701
	Body-site	590 755
	Host	1 166 464
	State	3 770 247
	Site	170 054
	Place	531 700
	Date	101 656
	Engineered	276 415
	Ecoregion	56 998
	Treatment	1 142 654
	Kit	100 791
	Primer	200 302
	Gene	396 448
	LS	135 940
	LCM	45 351
	Sequencing	120 520
ExTRI	Transcription factors-gene targets	22 552
IntAct	Protein–protein Interactions	323
PubTator	Gene mutations	1 222 576
PheneBank	Cells	6 512 005
	Gene mutations	713 445
	Phenotypes	25 729 643
	Molecules	26 033 617
	Anatomy	25 755 523
	Pathways	3 059 710
NaCTEM	Biological events (phosphorylation)	8766
OntoGene	Cells	9 739 164
	Cell lines	16 562 203
	Clinical drugs	19 121 812
	Sequences	13 236 865
	Molecular processes	1 607 426
	Organ/tissues	46 088 968
Open Targets Platform and DisGeNet	Gene–disease associations	20 998 732
SIB	Gene function (GeneRIF) statements	926 655
	COVoc	13 259 600

In 2021 the list of available annotations in Europe PMC was extended in response to the coronavirus pandemic, through a partnership with the Swiss Institute of Bioinformatics, to include the Coronavirus Vocabulary (COVoc) terms (Table 3) (10). These annotations are based on the COVoc ontology containing terms related to COVID-19 research, including pathogenicity, barrier gestures, treatments and more. COVoc ontology was developed to support literature triage for COVID-19. Articles with COVoc annotations can be found using the search syntax (ANNOTATION_TYPE:‘COVoc’) to help researchers and biological curators navigate scientific literature on COVID-19 (https://europepmc.org/AnnotationsApi).

Since the last update, Europe PMC expanded upon available annotations to recognise key metagenomics terms. A total of 16 novel metagenomics entities (Table 3), covering biome and experimental data, are identified from relevant full text publications using a machine learning framework, developed as part of the collaborative EMERALD project between Europe PMC and MGnify (https://www.ebi.ac.uk/metagenomics) (11). Publications with metagenomics annotations can be found in Europe PMC using the search syntax (Annotation_PROVIDER:’Metagenomics’). The metagenomics annotations pipeline is publically available via GitLab (https://gitlab.com/maaly7/emerald_metagenomics_annotations) and on bio.tools (https://bio.tools/emerald_metagenomics_annotations_pipeline), along with training datasets and machine learning models developed in the course of this project. Resulting annotations are used to enrich metagenomics datasets in the MGnify database with additional metadata on an ongoing basis.

To improve annotation accuracy and precision Europe PMC has explored the use of machine learning and deep learning techniques. Machine learning algorithms can help overcome some of the challenges of dictionary-based text-mining, but require gold standard datasets to train the machine learning models. For this purpose Europe PMC has created a set of 300 open access full text articles annotated with gene/protein, disease, organism and chemical bioentities identified by a team of expert human curators (12). The training sets are freely available at: https://gitlab.ebi.ac.uk/literature-services/public-projects/europepmc-corpus, in the formats and file types shown in Table 4. Available code includes scripts to clean and format annotations in Hypothes.is platform (https://web.hypothes.is/). The Europe PMC Annotations Corpus is one of the largest human-annotated publicly available gold standard biomedical corpora. It provides an open, extensive and accurate dataset, which can be used to improve the accuracy and reliability of life science natural language processing tools to support advancements in the field. For example, Open Targets, a public-private partnership for systematic drug target identification and prioritisation, uses the Europe PMC Annotations Corpus to identify relations between genes, diseases and drugs in published articles to provide evidence for target identification, validation and development (https://www.embl.org/news/science/machine-learning-to-identify-and-prioritise-drug-targets/).

**Table 4.** Data records for the Europe PMC Annotations Corpus including format and file type available

Format available	File type available
Stand-alone curator annotations	CSV
	JSON
	Inside-outside-beginning (IOB)
Full text of the article without Europe PMC annotations	XML
Full text of the article with sentence boundary (contains < SENT > tag to annotate the sentence boundary)	XML
Europe PMC annotations	JSON
	CSV
	IOB

To support literature analyses, Europe PMC makes open access content available for bulk download on the dedicated FTP site (https://europepmc.org/downloads). This includes the full text, supplementary data files and figures for open access publications and preprints, author manuscript collection, metadata for full text Europe PMC articles, DOI–PMCID–PMID mappings and text-mined accession numbers from major life sciences databases.

To address user requests Europe PMC introduced a bulk download for the PDF files of full text publications in the open access subset, in addition to the existing XML download option. Over 5.7 million articles in this subset are made available under a Creative Commons licence or similar, which enables redistribution and reuse. The directory is updated weekly and archived quarterly.

As an open science database, Europe PMC is committed to providing rich metadata records. One area of metadata improvements was the addition of ROR IDs (https://ror.org/) to grant records in the Europe PMC Grant Finder tool (https://europepmc.org/grantfinder/). ROR IDs are persistent identifiers for research organisations that help disambiguate names for over 102 000 institutions. For example, the University of Cambridge may also be referred to as Cambridge University, or UoC and more variations. To improve grant metadata records and affiliation search of the Grant Finder tool we have mapped institutions in the Europe PMC grants database (GRIST) to corresponding ROR IDs. Some of the Europe PMC funders already supply ROR IDs in the data they provide, however this only accounted for 10% of the grants in Europe PMC. We used an automated script (https://gitlab.ebi.ac.uk/literature-services/public-projects/ror-api-matching-script) to identify matching ROR IDs for institutional names using the ROR API (https://ror.readme.io/docs/rest-api), followed by a manual checking step. This resulted in 41% of institutions confidently matched and over 80% of grants in Europe PMC being linked to the principal investigator's institutional ROR ID. Enriching the grants metadata available in Europe PMC enabled further improvements to the Grant Finder tool, consolidating different institutional aliases under one ROR ID for the auto-suggestion feature. Grant information, including associated ROR IDs, can be retrieved programmatically using GRIST API core response (https://europepmc.org/GristAPI).

## Tools to keep up to date with research

### Article Status Monitor

**Figure 3.**
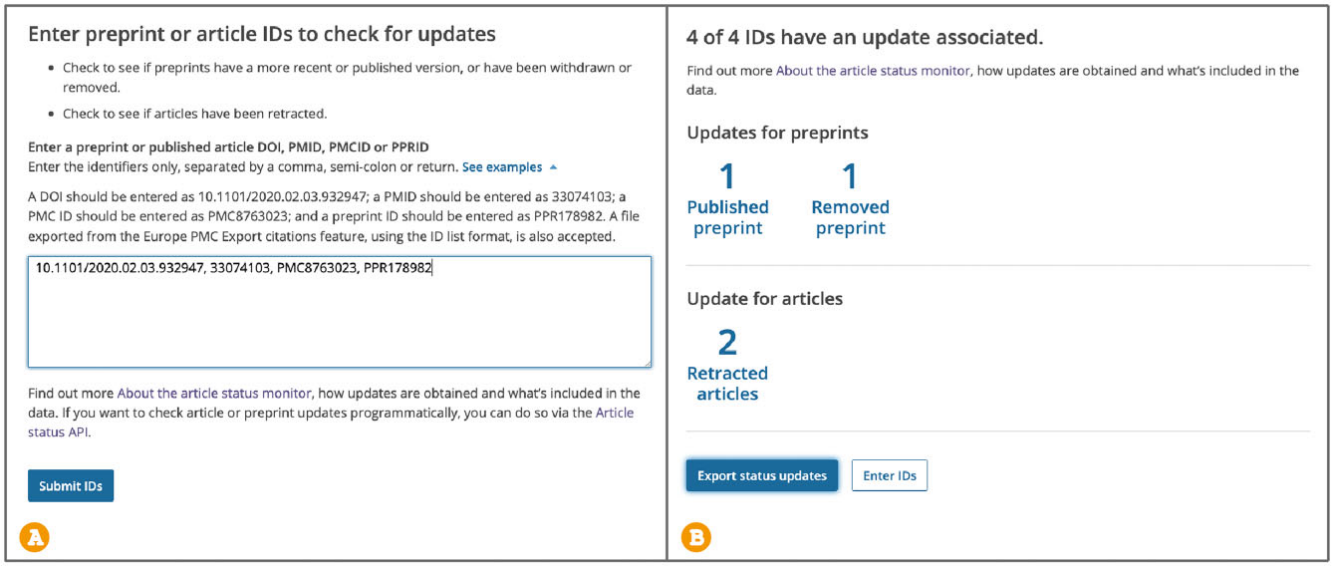
Article Status Monitor dashboard: (A) the view to input preprint or article IDs; (**B**) returned updates for the IDs searched (10.1101/2020.02.03.932947, 33074103, PMC8763023, PPR178982).

When working with scientific literature, researchers need to know whether conclusions still stand after a retraction or withdrawal, which preprint version to cite, or what changes were made to the published version. To help users retrieve status updates for publications and preprints Europe PMC developed the Article Status Monitor tool (6). This tool allows users to check if a preprint has been withdrawn or removed, published in a journal, or updated with a more recent version, or whether a journal article has been retracted. The Article Status Monitor accepts various publication identifiers (IDs), including DOIs, PMIDs, PMCIDs or Europe PMC preprint identifiers (PPRIDs). It is also possible to input a list of identifiers or upload an ID list file generated with the ‘Export citations’ feature. Information about available status updates is displayed on the Articles Status Monitor dashboard (Figure 3). Users can follow a link to the updated record on the Europe PMC website or export status updates as a CSV file containing publication IDs for the original and updated record, as well as status update type. The data on status updates for the Article Status Monitor is obtained through a variety of methods. Information about preprint withdrawal or removal is available for COVID-19 and Europe PMC funder preprints through semi-automated analysis of the preprint full text. Currently updates on withdrawals and removals are unavailable for abstract-only preprints in Europe PMC, as there is no corresponding metadata provided in Crossref. For preprints published in a peer-reviewed journal the Articles Status Monitor uses available Crossref metadata alongside an algorithm to match preprint titles and author details to peer-reviewed journal articles indexed in Europe PMC. Note that in some cases, where authors and/or article titles change between preprint and journal publication, it may not be possible to make this connection. Updates on preprint versions are available for records from preprint servers that issue separate DOIs for new preprint versions. Where the same DOI is used for different versions, for example in the case of bioRxiv or medRxiv, Europe PMC cannot link the versions to each other. Finally, Europe PMC receives journal article retraction notices provided by the publishers from MEDLINE and links them to the relevant article. The Article Status Monitor tool can also be accessed programmatically using the /status-updates-search module of the Articles RESTful API (https://europepmc.org/RestfulWebService). To retrieve status updates for selected publication IDs, users need to make a POST request with the header Content-type:application/json, passing all parameters in the request body as a JSON object. POST search requests should be constructed as follows: POST https://www.ebi.ac.uk/europepmc/webservices/rest/status-update-search with request body { ids: [ { src: string, extId: string}]}. Available response formats include XML and JSON.

### Email alerts for keyword searches

**Figure 4.**
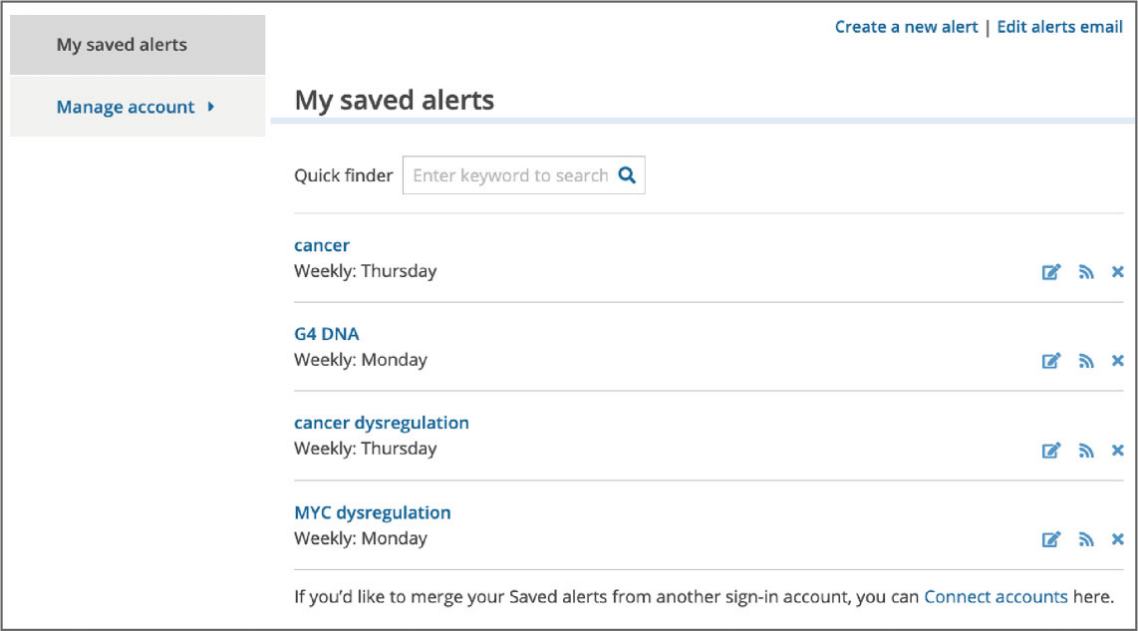
Europe PMC user account, ‘My saved alerts’ page.

**Figure 5.**
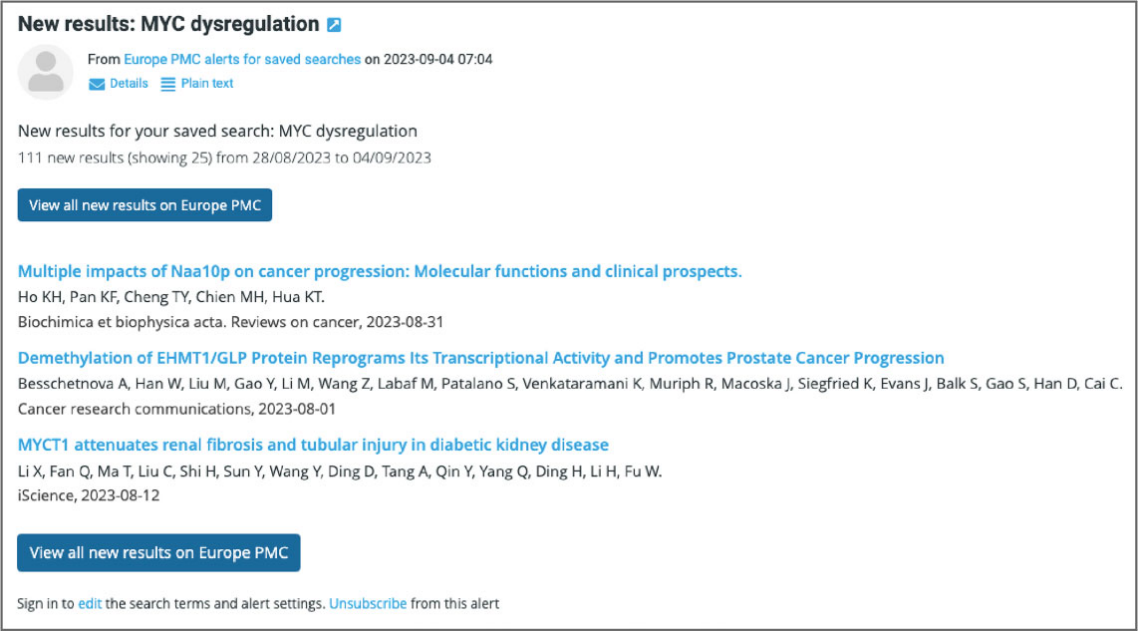
Email alert from Europe PMC for the saved search (MYC dysregulation). Details for search results include title, author(s) name(s) and journal information, with the option to view all search results in Europe PMC. An ’Unsubscribe’ option is available at the bottom of the email.

To notify users about new publications relevant to their research, Europe PMC introduced email alerts. Personalised alerts can be set for any keyword search in Europe PMC using the ‘Save & create alert’ button. To set up an alert, users need to create a Europe PMC account by signing in with their email, ORCID, Twitter, or Europe PMC plus account. All of the saved alerts can be accessed, modified or deleted from the user account page at any time (Figure 4). Alerts can be customised to meet the user's needs, for example by choosing the frequency of email updates or publication details included in the email. Users can opt to receive updates as soon as they become available, or choose between weekly or monthly options. It is also possible to save the search without subscribing to updates, to run it manually at the user's convenience. Email alerts include title, author(s) name(s), journal information and partial abstract (if selected) for each publication. Details for up to 25 search results are shown in the email, with an option to view all new search results in Europe PMC (Figure 5). It is also possible to subscribe for search updates using custom RSS feeds. Users can add the feed for the first 25 Europe PMC search results by selecting the ‘Subscribe to RSS’ option on the search results page. The new email alerts feature helps users save time and stay on top of the current literature. Alerts can be used to track topics of interest, as well as articles from particular authors, research groups, institutions, or journals. Since Europe PMC offers comprehensive coverage for biomedical preprints, this feature can also be used to learn about new preprints from 31 preprint servers. As alerts can be set up for any keyword search, users can benefit from the many advanced search options that Europe PMC provides. For example, an email alert for the *CITES:(publication ID_publication source)* search can notify users about new works citing their publication. It is also possible to follow new citations for datasets from over 60 life science databases using the *ACCESSION_ID:(data accession number)* search. This is a unique feature of Europe PMC that supports data reuse. Alerts for more advanced search options can be constructed using the search syntax reference on the Europe PMC website (https://europepmc.org/searchsyntax).

### Europe PMC as an open scholarly infrastructure

As a community driven, sustainable, open infrastructure, Europe PMC adopted the principles of open scholarly infrastructure (POSI) in 2022 (https://blog.europepmc.org/2022/02/europe-pmc-adopts-the-principles-of-open-scholarly-infrastructure.html). Following a self audit exercise Europe PMC has increased the proportion of open source core software, and committed to developing all new code bases as open source projects. For example, the code for Europe PMC text-mining API, which can be used for tagging bioentities, is now open source and can be accessed here: https://gitlab.ebi.ac.uk/literature-services/public-projects/textmining-public-api. Furthermore, we have shared the code for a docmap-parser written in Scala programming language here: https://gitlab.ebi.ac.uk/literature-services/public-projects/docmap-parser. The parser can transform DocMaps files from JSON-LD into XML format. Europe PMC uses this as an important step in ingesting preprint peer review materials from Sciety into Europe PMC. This code may be useful for other users that need to perform a similar transformation step for importing DocMaps data into different systems. All of the Europe PMC open source code can be found on a public GitLab page: https://gitlab.ebi.ac.uk/literature-services/public-projects.

Another essential part of the POSI guidelines is service sustainability. Long term stability of Europe PMC is important to provide a permanent, freely available, digital archive of life science research outputs. In support of the vital role of Europe PMC it was recently recognised as a data resource critical to life science research worldwide by the Global Biodata Coalition, demonstrating the importance of continued funding for Europe PMC (https://globalbiodata.org/what-we-do/global-core-biodata-resources/list-of-current-global-core-biodata-resources/).

One of the core principles of open scholarly infrastructure is to provide free access to information. This includes making services accessible to all users regardless of their mode of access. In 2021, after a careful audit of the website pages, several improvements in website accessibility were made. We have ensured that website pages are keyboard accessible, and improved navigation for screen readers and braille displays to support users relying on voice and keyboard commands. We continue to monitor and improve website accessibility to meet guidelines set out by W3C accessibility standards (https://www.w3.org/WAI/standards-guidelines/).

## Concluding comments

As an open scholarly infrastructure provider, Europe PMC aims to support innovation and discovery and provide a world-class, reliable and high-performance service. Services are continually improved, focusing on good user experience as a major driving force. Since 2020 Europe PMC has had several major updates, focusing on preprints, new tools to track research literature, text-mining support, accessibility and open sourcing. We significantly increased the number of preprints in Europe PMC by indexing content from new servers and expanded the number of full text preprints to include those supported by Europe PMC funders. Our work to build trust in preprints centred around identifying withdrawn and removed content, and linking preprints to available reviews and evaluations. To support text-mining efforts we have developed a gold standard Annotations Corpus, which can be used to train machine learning models and support advancements in the field. Available text-mined annotations have been expanded to include new types. We have developed new tools for literature research, including the Article Status Monitor tool and email alerts. To improve trust in the sustainability of Europe PMC we have self-assessed against the principles of open scholarly infrastructure, increased the proportion of open source software for services, and committed to open source all new code bases developed. Europe PMC is now a Global Biodata Coalition Core Resource, as well as an ELIXIR Core Data Resource.

In the future, Europe PMC will focus on ingesting and displaying preprint peer reviews, and improving annotation accuracy and precision, using the Europe PMC Annotations Corpus to develop machine learning filters. To keep up to date with Europe PMC’s current and future projects visit the quarterly roadmap at: https://europepmc.org/Roadmap.

## Data Availability

Europe PMC services are freely available at (https://europepmc.org/). Europe PMC APIs and bulk downloads are freely available at (https://europepmc.org/developers).All of the Europe PMC open source code can be found on a public GitLab page (https://gitlab.ebi.ac.uk/literature-services/public-projects).Content is distributed under the EMBL-EBI Terms of Use available at (https://www.ebi.ac.uk/about/terms-of-use).

## Notes

Present address: Maaly Nassar, SciBite, an Elsevier Company, Wellcome Trust Genome Campus, Cambridge, UK.

Present address: Shyamasree Saha, Exscientia, the Schrödinger Building Oxford Science Park, Oxford, UK.
